# Space-time clustering of childhood central nervous system tumours in Yorkshire, UK

**DOI:** 10.1186/1471-2407-12-13

**Published:** 2012-01-13

**Authors:** Richard JQ McNally, Peter W James, Susan V Picton, Patricia A McKinney, Marlous van Laar, Richard G Feltbower

**Affiliations:** 1 Institute of Health and Society, Newcastle University, Sir James Spence Institute, Royal Victoria Infirmary, Newcastle upon Tyne NE1 4LP, UK; 2 Paediatric Oncology and Haematology, Leeds Teaching Hospitals NHS Trust, Beckett Street, Leeds LS9 7TF, UK; 3 Paediatric Epidemiology Group, Division of Epidemiology, University of Leeds, Leeds LS2 9NL, UK; 4 Dr Richard JQ McNally, Institute of Health and Society, Newcastle University, Sir James Spence Institute, Royal Victoria Infirmary, Queen Victoria Road, Newcastle upon Tyne NE1 4LP, UK

## Abstract

**Background:**

We specifically tested the aetiological hypothesis that a factor influencing geographical or temporal heterogeneity of childhood central nervous system (CNS) tumour incidence was related to exposure to a transient environmental agent.

**Methods:**

Information was extracted on individuals aged 0-14 years, diagnosed with a CNS tumour between the 1st January 1974 and 31st December 2006 from the Yorkshire Specialist Register of Cancer in Children and Young People. Ordnance Survey eight-digit grid references were allocated to each case with respect to addresses at the time of birth and the time of diagnosis, locating each address to within 0.1 km. The following diagnostic groups were specified *a priori *for analysis: ependymoma; astrocytoma; primitive neuroectodermal tumours (PNETs); other gliomas; total CNS tumours. We applied the *K*-function method for testing global space-time clustering using fixed geographical distance thresholds. Tests were repeated using variable nearest neighbour (NN) thresholds.

**Results:**

There was statistically significant global space-time clustering for PNETs only, based on time and place of diagnosis (*P *= 0.03 and 0.01 using the fixed geographical distance and the variable NN threshold versions of the *K*-function method respectively).

**Conclusions:**

There was some evidence for a transient environmental component to the aetiology of PNETs. However, a possible role for chance cannot be excluded.

## Background

Central nervous system (CNS) tumours are the second most common childhood malignancy in resource-rich countries [1]. Aetiology is poorly understood, but is likely to involve both genetic and environmental factors. Putative environmental factors have been identified from case-control studies including maternal consumption of cured meats, fish, tea and coffee whilst pregnant, N-nitroso compounds, exposure to insecticides or pesticides, animals and farm life, electro-magnetic fields and lack of social contact during the first year of life [2-7]. A role for infectious agents has been postulated [8]. If infections that are non-ubiquitous and non-endemic are involved in disease aetiology, then among cases the distribution of births (for an early exposure) or diagnoses (for a later exposure) may exhibit seasonal variation and/or space-time clustering. This would only occur when the latent period from exposure to birth or diagnosis is short or at least reasonably constant. The onset of a tumour would result as a rare response to an infection. Both seasonal variation and space-time clustering are also consistent with the involvement of any other transient environmental exposure in aetiology.

A number of recent studies have found seasonal variation. One study from Japan identified a peak for all central nervous system tumours who were born in the winter [9], a study from northern England found an excess of astrocytoma cases born in October [10], and a study from the USA showed an excess of PNET (medulloblastoma, NOS) cases born in October [11]. There have also been several recent studies of space-time clustering of national (GB) childhood cancer data [12,13], which found statistically significant evidence of clustering overall and marginally significant evidence for cases of astrocytoma diagnosed during the period 1969-1993. A study from North West England found evidence of space-time clustering and seasonal variation amongst cases of childhood CNS tumours, particularly astrocytoma and ependymoma [14]. Another study from the Netherlands of adult gliomas, diagnosed in cases aged more than 15 years, found evidence of overall space-time clustering which could not be attributed to a specific sub-type [15,16].

We have analysed incidence data from the population-based Yorkshire Specialist Register of Cancer in Children and Young People (YSRCCYP). The analyses are based on both the address at birth and the address at diagnosis. The present study updates and extends previous analyses of data from earlier time periods. It also extends previous analyses by studying individual diagnostic groups and allows a comparison to be made between clustering that may have arisen from a geostationary exposure and clustering that may have originated from an infective source.

The first aim was to test predictions of space-time clustering occurring among childhood CNS tumours which might arise as a result of environmental causal mechanisms. The second aim was to distinguish between clustering that may be related to a geostationary source from clustering that may have an infectious origin. The third aim was to identify specific space-time clusters and to test for differences between 'clustered' and 'non-clustered' cases. We specifically tested the aetiological hypothesis that a factor influencing geographical or temporal heterogeneity of childhood CNS tumour incidence was related to exposure to a transient environmental agent.

### Prior hypotheses

The following aetiological hypotheses were tested: (i) a primary factor influencing geographical or temporal heterogeneity of incidence of childhood CNS tumours was related to exposure to a transient environmental agent; (ii) geographical or temporal heterogeneity of incidence of childhood CNS tumours was modulated by differences in susceptibility between males and females; and (iii) geographical or temporal heterogeneity of incidence of childhood CNS tumours was modulated by differences in patterns of exposure related to level of population density.

## Methods

### Study subjects

Information on all cases of CNS tumours in 0-14 year olds diagnosed in the former Yorkshire Regional Health Authority during the period 1st January 1974 to 31st December 2006 was extracted from the Yorkshire Specialist Register of Cancer in Children and Young People (YSRCCYP) [17]. The YSRCCYP is a specialist population-based cancer registry covering an area of 12,000 km^2 ^which varies between highly urbanised conurbations such as Leeds and Bradford in West Yorkshire to rural isolated areas such as the North York moors in North Yorkshire. The socio-demographic profile of the Yorkshire region has been shown to be representative of the UK as a whole [18]. The YSRCCYP is exempted (originally under Section 60 of the UK Health and Social Care Act 2001, which has now been superseded by Section 251 of the National Health Service Act 2006) from the need to obtain patient consent for recording and analysis of data. The original ethical approval for the YSRCCYP was granted by the Northern and Yorkshire Research Ethics Committee in April 2000 (reference MREC 0/3/1) which allows epidemiological research, including space-time clustering, to be conducted using Register data.

Cases were ascertained from hospital clinics and neuropathology departments across the Region, and further validation checks for completeness were carried out with the National Registry of Childhood Tumours (http://www.ccrg.ox.ac.uk) and the Northern and Yorkshire Cancer Registry and Information Service (http://www.nycris.org.uk). 85% of all diagnoses recorded on the YSRCCYP have been histologically verified and a case review (undertaken by a single experienced neuropathologist) of all CNS tumours on the Register was carried out in 2004 to validate tumour classification [19].

Malignant or certain benign CNS tumours were included in the analysis occurring within Group III of the International Classification of Childhood Cancer (ICCC) based on ICD-O-2 morphology and site codes [20]. The following diagnostic groups were specified *a priori *for analysis: (i) ependymoma (ICCC code III(a)); (ii) astrocytoma (ICCC code III(b)); (iii) ependymoma and astrocytoma (ICCC codes III(a) and III(b)); (iv) PNET (ICCC code III(c)); (v) other gliomas, e.g. oligodendroglioma, mixed glioma, other glioma situated outside the optic nerve (ICCC code III(d)); (vi) other specified and unspecified CNS tumours (ICCC codes III(e&f)); and (vii) all CNS tumours (ICCC codes III(a-f)) [21]. All CNS tumours, except intracranial germ cell tumours, are captured by the ICCC IIIa-f codes. Benign tumours included cases of ependymoma, other gliomas, other specified intracranial and intraspinal neoplasms and unspecified intracranial and intraspinal neoplasms.

In the UK there are around 1.7 million postcodes, which are primarily used for postal delivery. A typical postcode may include around fifteen to twenty houses, a smaller number of multiple occupancy residences, or a single commercial address [22]. For each case, Ordnance Survey (OS) four-digit Easting and Northing grid references were allocated to the centroid of the birth and diagnosis residential address postcode. This allowed geo-referencing of the Easting and Northing residential address co-ordinates to within 0.1 km.

### Statistical methods

Overall space-time clustering was studied using an approach based on *K*-functions, which may be considered to be a generalised version of the Knox test [23,24]. These methods have been used in previous work related to space-time clustering of childhood cancer, type 1 diabetes and congenital anomalies [12,13,25,26]. The Knox test regards a pair of cases as being in "close proximity" if diagnosis time and addresses of residence at this time are close. The number of pairs of cases observed to be in close proximity is counted and denoted *O*. The number of pairs of cases expected to be in close proximity, assuming independence of spatial and temporal proximity, is calculated and denoted *E*. If *O *is greater than *E*, then a significance test is used to determine if there is evidence of space-time clustering. An estimate of the "strength of clustering" is obtained by calculating $S=\left(\frac{\left[O-E\right]}{E}\right)\times 100$. A related quantity is defined as *R *= O/√E.

The Knox test presents a particular limitation, namely the choice of critical values is entirely arbitrary. This test uses a single set of critical values for defining close proximity in space and time (e.g. "close in space", denoted *s *= 1 km, and "close in time", denoted *t *= 12 months). Selection of a number of different critical values and subsequent repetition of the Knox analysis would result in multiple testing. A simplification of the *K*-function method has been used to partially avoid the arbitrary choice of critical values and therefore avoid multiple testing [23]. This approach involved a simultaneous set of 225 calculations similar to the single Knox calculations to obtain values of *R*. Critical values changed over a pre-specified set of close values in time (*t *= 0.1, 0.2,...,1.5 years) and close values in space (*s *= 0.5, 1.0, 1.5,..., 7.5 km). The observed value of the *K*-function, *K_O_*, was obtained by summing the 225 calculated values of *R(s,t)*, i.e. *K_O _*= ∑*_s,t_R *and the distribution of the *K*-function was simulated using 999 random permutations of time. At each simulation, dates of birth (or dates of diagnosis) were randomly reallocated to each of the cases in the data set, creating a simulated value of the *K*-function. Note that the Knox test corresponds to a single dimension *K*-function where there is only one set of critical values. Statistical significance was assessed by comparing the observed value of the *K*-function with the simulated distribution.

Unlike the Knox test, the *K*-function does not give a readily available measure of the size of the clustering effect. Hence *S *(obtained from the Knox test, with critical spatial values *s *= 0.5,...,7.5 km and critical temporal values *t *= 0.1,...,1.5 years) was used to describe the magnitude of the clustering effects for a given pair of critical values. Additionally, the nominal statistical significance of each value of *S *was assessed using the Poisson distribution. To enable comparisons to be made between the geographical distance and nearest neighbour (NN) metrics (see below), an overall indicator of the strength of clustering was obtained using $\sum_{i=1}^{225}S$ (where i refers to the *i^th ^*combination of *s *and *t*).

If clustering has arisen due to a geostationary exposure, then this could lead to detection only by the fixed geographical distance threshold. Alternatively, if clustering has arisen due to an infective process, then this could lead to detection only by the variable NN threshold. If clustering is due to an infective process, then it must be noted that analysis based on a NN metric is likely to be more appropriate when both urban and rural areas are included. Any specified distance between two cases will have different meanings in urban and rural locations. For example, the size of school catchment areas will differ greatly. Using the NN metric the specification of critical values for "close in space" is not fixed, but determined empirically by the local density of the spatially heterogeneous underlying population. Using the *n^th ^*NN, two cases were close in space if the locations of one (or both) of the cases was nearer than the other's *n^th ^*NN in the total data set (of all birth and diagnosis addresses). Thus the number of these pairs of cases observed to be in close proximity was counted. To adjust for variations in population densities, we repeated the *K*-function analyses by replacing fixed geographical distances with variable distances to the (*N*-7)*^th^*,...,(*N *+ 7)*^th ^*NNs if *N *≥ 8 and with variable distances to the 1st,..., 15th NNs if *N *≥ 7. *N *was chosen so that the mean distance was around 5 km, thus *N *= 3 for birth addresses (the fixed geographical distances were replaced by variable distances to the 1st,...,15th NNs) and *N *= 12 for diagnosis addresses (the fixed geographical distances were replaced by variable distances to the 5th,...,19th NN). The use of a single threshold NN approach was originally proposed by Jacquez [27].

The distributions of distances between the 3rd NNs for births and the 12th NNs for diagnoses were highly skewed, with median distances of 1.2 km and 2.9 km respectively. An exact geographically based match to the underlying population distribution was not available. Thus we used the case distribution as a proxy for the underlying population distribution to test whether population density was associated with space-time clustering. Cases were divided into two groups: 50% in a "more densely populated" group and 50% in a "less densely populated" group, according to whether the 3rd NN (for births) or 12th NN (for diagnoses) was closer or further away than the median distance. There are then three possible ways in which pairs of cases may be in close proximity: (i) a case from a "more densely populated" area may be in close proximity to another case from a "more densely populated" area; (ii) a case from a "less densely populated" area may be in close proximity to another case from a "less densely populated" area; or (iii) a case from a "more densely populated" area may be in close proximity to a case from a "less densely populated" area. Therefore, if we are interested in whether cases from a "more densely populated" area show a tendency to cluster, it does not matter whether partner cases are from either a more or less densely populated area. Thus, population density analyses proceeded by analysing pairs of cases that included at least one case from a "more densely populated" area (i.e. "more densely populated: any" case pairs) and pairs of cases that included at least one case from a "less densely populated" area (i.e. "less densely populated: any" case pairs).

It has been argued that population shifts may cause artificial space-time clustering [28,29]. We were not able to analyse population shifts, because this would require data on small area population estimates for short time intervals, which are not available. If population shifts led to space-time clustering we would predict that this would only occur within a specific sub-period. Thus, we also analysed space-time clustering within two shorter time periods (1974-1990 and 1991-2006).

As a supplementary analysis, Kulldorff's scan statistic based on a space-time permutation model was used to identify individual clusters [30] and examine geographical and spatial patterning between covariates (and thus this method is distinct from the Knox and *K*-function methods which analyse overall space-time clustering patterns). The complete study region and time span was scanned by construction of a three-dimensional cylindrical moving window. The base of the cylinder represents two-dimensional geographical space and the height represents time. The base and height of this cylinder vary so that they include at most 10% of the entire time span and at most 10% of the entire geographical area. The variable base is centred on the postcode centroid of each case [31]. This method has been used previously in an analysis of childhood leukaemia data [32]. The scan statistic was applied to test for differences in the propensity to cluster between gender and levels of population density, using a Bernoulli-based model [33]. This method is a case-control approach where one stratum (e.g. males) is treated as the case group and the other stratum (e.g. females) is treated as the control group. Thus the test assesses differences between the spatio-temporal distributions of the two groups. These scan statistics were calculated using the geographical locations of the addresses (OS grid references of residence at birth or diagnosis) and temporal reference (date of birth or date of diagnosis).

Four possible space-time interactions were analysed: those between (i) times and places of birth; (ii) time of diagnosis and place of birth; (iii) time of birth and place of diagnosis; and (iv) times and places of diagnosis. The interpretation of these interactions depends on the extent of residential movement between birth and diagnosis among the cases. If there was no residential movement then there would only be two interactions (time of birth or diagnosis with place of domicile). An interaction based on birth would indicate that cases who resided close to one another were also born at close points in time, indicating that they shared a similar environment at birth. An interaction based on diagnosis would indicate that cases who resided close to one another were also diagnosed at similar times, suggesting that they shared a similar environment at diagnosis. However, more than approximately 60% of children moved between birth and diagnosis, indicating that residential movements need to be taken into account. Thus a time of birth/place of birth interaction would suggest a transient environmental exposure affecting children in-utero or shortly after birth and that there is a variable latent period between exposure and diagnosis. A time of diagnosis/place of diagnosis interaction would suggest an exposure around diagnosis place and close to diagnosis time with a short latent period. A time of diagnosis/place of birth interaction would indicate an exposure at a heterogeneous time after birth, with a constant latent period. A time of birth/place of diagnosis interaction would suggest an exposure around residence at diagnosis, affecting those born at similar times with a short latent period (for a more detailed description see Birch and colleagues [34]). *K*-function and Knox analyses were done using programs written in FORTRAN 90 [35] and Kulldorff's scan statistic was performed using SaTScan v7.0 [36].

Statistical significance (*P *< 0.05) was evaluated using one-sided tests and 999 simulations for both the *K*-function analyses and the scan statistic.

## Results

Details of 693 cases diagnosed between 1974 and 2006 were extracted from the YSRCCYP. Of these, 506 (73%) had birth address details and this proportion was consistent across all diagnostic groups. Table 1 shows the number of cases by diagnostic sub-group and gender.

**Table 1:** Numbers of children by disease group, with a diagnosis address in the region and with a birth address (diagnosed in the region) in the region

ICCC^a ^code(s)	Disease group	Number with diagnosis address in region	Number with birth address (diagnosed in the region)
III(a)	Ependymoma	59 (M^b ^= 39; F^c ^= 20)	46 (M = 32; F = 14)

III(b)	Astrocytoma	293 (M = 148; F = 145)	214 (M = 109; F = 105)

III(a), III(b)	Ependymoma and Astrocytoma	352 (M = 187; F = 165)	260 (M = 141; F = 119)

III(c)	PNET	165 (M = 103; F = 62)	120 (M = 77; F = 43)

III(d)	Other gliomas	79 (M = 46; F = 33)	58 (M = 33; F = 25)

III(e), III(f)	Other Specified and Unspecified	97 (M = 48; F = 49)	68 (M = 34; F = 34)

III(a-f)	All CNS tumours	693 (M = 384; F = 309)	506 (M = 285; F = 221)

^a^International Classification of Childhood Cancer.

^b^Males.

^c^Females.

There was no evidence of overall space-time clustering based on place of birth and date of birth for all CNS tumours or for any diagnostic sub-group (Table 2). There was also no evidence of overall space-time clustering based on place of birth and date of diagnosis (Table 3), nor based on place of diagnosis and date of birth (Table 4).

**Table 2:** Results of *K*-function space-time clustering analyses for place of birth and date of birth^a^

ICCC^b ^Code(s)	Disease group	Geographical distance^c ^(*P*-values)	NN threshold^d ^(*P*-values)
III(a)	Ependymoma	^e^	0.49

III(b)	Astrocytoma	0.79	0.49

III(a), III(b)	Ependymoma and Astrocytoma	0.88	0.41

III(c)	PNET	0.42	0.52

III(d)	Other gliomas	0.52	0.17

III(e), III(f)	Other Specified and Unspecified	0.37	0.34

III(a-f)	All CNS tumours	0.10	0.14

^a^Cases are close in time if dates of birth differ by <*t*, where *t *is in the range 0.1 year to 1.5 year.

^b ^International Classification of Childhood Cancer.

^c^Cases are close in space if distances between their locations differ by <*s*, where *s *is in the range 0.5-7.5 km.

^d^Cases are close in space if either one is within the distance to the *N^th ^*nearest neighbour of the other, where *N *ranges from 1-15.

^e^Too few cases for analysis

**Table 3:** Results of *K*-function space-time clustering analyses for place of birth and date of diagnosis^a^

ICCC^b ^code(s)	Disease group	Geographical distance^c ^(*P*-values)	NN threshold^d ^(*P*-values)
III(a)	Ependymoma	^e^	0.51

III(b)	Astrocytoma	0.26	0.46

III(a), III(b)	Ependymoma and Astrocytoma	0.41	0.77

III(c)	PNET	0.10	0.21

III(d)	Other gliomas	0.43	0.39

III(e), III(f)	Other Specified and Unspecified	0.85	0.36

III(a-f)	All CNS tumours	0.53	0.48

^a^Cases are close in time if dates of diagnosis differ by <*t*, where *t *is in the range 0.1 year to 1.5 year.

^b ^International Classification of Childhood Cancer.

^c^Cases are close in space if distances between their locations differ by <*s*, where *s *is in the range 0.5-7.5 km.

^d^Cases are close in space if either one is within the distance to the *N^th ^*nearest neighbour of the other, where *N *ranges from 1-15.

^e^Too few cases for analysis.

**Table 4:** Results of *K*-function space-time clustering analyses for place of diagnosis and date of birth^a^

ICCC^b ^code(s)	Disease group	Geographical distance^c ^(*P*-values)	NN threshold^d ^(*P*-values)
III(a)	Ependymoma	^e^	0.73

III(b)	Astrocytoma	0.75	0.63

III(a), III(b)	Ependymoma and Astrocytoma	0.94	0.87

III(c)	PNET	0.69	0.42

III(d)	Other gliomas	0.85	0.82

III(e), III(f)	Other Specified and Unspecified	0.53	0.26

III(a-f)	All CNS tumours	0.74	0.40

^a^Cases are close in time if dates of birth differ by <*t*, where *t *is in the range 0.1 year to 1.5 year.

^b ^International Classification of Childhood Cancer.

^c^Cases are close in space if distances between their locations differ by <*s*, where *s *is in the range 0.5-7.5 km.

^d^Cases are close in space if either one is within the distance to the *N^th ^*nearest neighbour of the other, where *N *ranges from 1-15.

^e^Too few cases for analysis.

For the analyses based on place of diagnosis and date of diagnosis there was evidence of statistically significant overall space-time clustering for the group comprising PNET (ICCC code III(c)) only (*P *= 0.03, and *P *= 0.01, using the geographical distance and NN threshold versions of the *K*-function method, respectively; see Table 5). The strength of clustering (*S*) of PNET was summed over all 225 combinations of space and time ($\sum_{i=1}^{225}S$) and was calculated as 13819 using the geographical distance and 17177 using the NN versions of the Knox test, respectively. For 177 of 225 combinations of space and time, *S *was greater using the NN version of the method compared with the geographical distance version. Thus, space-time clustering was more marked using the NN metric.

**Table 5:** Results of *K*-function space-time clustering analyses for place of diagnosis and date of diagnosis^a^

ICCC^b ^code(s)	Disease group	Geographical distance^c ^(*P*-values)	NN threshold^d ^(*P*-values)
III(a)	Ependymoma	0.64	0.81

III(b)	Astrocytoma	0.31	0.14

III(a), III(b)	Ependymoma and Astrocytoma	0.67	0.62

III(c)	PNET	0.03^e^	0.01^e^

III(d)	Other gliomas	0.26	0.55

III(e), III(f)	Other Specified and Unspecified	0.79	0.85

III(a-f)	All CNS tumours	0.84	0.92

^a^Cases are close in time if dates of diagnosis differ by <*t*, where *t *is in the range 0.1 year to 1.5 year.

^b ^International Classification of Childhood Cancer.

^c^Cases are close in space if distances between their locations differ by <*s*, where *s *is in the range 0.5-7.5 km.

^d^Cases are close in space if either one is within the distance to the *N^th ^*nearest neighbour of the other, where *N *ranges from 1-15.

^e^Statistically significant (*P *< 0.05).

Analyses by two shorter time periods (cases diagnosed between 1974 and 1990; and cases diagnosed between 1991 and 2006) found that overall space-time clustering was still present in both of these time periods (for cases diagnosed between 1974 and 1990: *P *= 0.24 using the geographical distance and *P *= 0.03 using the NN versions of the *K*-function method; and for cases diagnosed between 1991 and 2006: *P *= 0.41 using the geographical distance and *P *= 0.04 using the NN versions of the *K*-function method respectively).

For PNETs, the strength of clustering (calculated using the geographical distance version of the Knox method) was positive for most critical values. It was most marked for cases diagnosed within 0.1 to 0.2 years (1-3 months) of one another. However, nominally statistically significant values were mainly confined to two spatial bands (0-2 km and 4.5-5 km) and to cases that were diagnosed between 0.9 and 1.5 years of one another (Table 6). The Knox test showed that there were a number of small sized case aggregations (where an individual case had at most 3 other cases in close spatiotemporal proximity) using critical values for space and time of 5 km and 12 months, respectively.

**Table 6:** Strength (*S*) obtained from clustering results, for date and place of diagnosis, for PNET sub-group (ICCC^a ^code III(c)) from Knox tests with a range of critical values for space and time^b^

Time (year)	Distance (km)														
	**0.5**	**1**	**1.5**	**2**	**2.5**	**3**	**3.5**	**4**	**4.5**	**5**	**5.5**	**6**	**6.5**	**7**	**7.5**

0.1	-100.0	373.7	155.7	239.1	136.9	66.9	32.0	53.9	23.0	40.4	24.1	10.9	48.5	59.3	66.7

0.2	-100.0	148.7	168.5	167.0	148.7	75.3	38.6	34.7	50.6	47.4	30.4	16.4	29.9	31.4	42.2

0.3	-100.0	57.3	69.8	68.9	57.3	10.8	-12.3	2.2	22.5	16.5	3.0	1.3	6.8	13.3	17.6

0.4	237.6	257.4	157.2	113.2	108.5	46.9	16.2	16.1	34.0	32.4	17.1	11.6	18.2	20.2	20.5

0.5	172.3	188.4	107.5	72.0	68.2	52.4	20.6	24.9	33.1	35.3	25.9	18.1	20.5	20.0	18.4

0.6	357.4	**222.9**	117.8	73.3	61.4	42.2	12.5	13.6	25.7	31.5	26.9	18.1	22.3	24.0	20.7

0.7	290.8	175.9	123.3	97.5	72.4	58.0	24.9	27.0	31.3	37.9	31.0	21.1	22.5	22.5	18.3

0.8	239.1	139.4	93.8	71.3	49.6	37.0	16.7	36.1	34.6	**41.8**	33.3	26.1	28.1	26.4	21.1

0.9	**361.5**	**171.5**	**134.4**	**94.3**	62.9	43.5	28.6	**47.0**	**40.9**	**44.8**	**35.2**	27.1	27.6	25.1	19.4

1.0	**319.7**	146.9	**113.2**	76.7	48.1	30.5	17.0	39.0	32.4	**35.3**	26.1	18.5	18.6	16.2	10.8

1.1	**280.5**	123.8	93.3	76.2	45.5	26.2	18.5	35.7	31.7	**32.6**	23.2	17.9	16.9	13.9	8.3

1.2	252.3	**190.2**	**123.7**	**92.9**	55.5	31.4	21.3	34.7	**36.3**	**38.2**	27.6	21.3	19.1	15.5	9.4

1.3	224.0	**166.8**	**126.3**	**91.0**	52.5	27.6	16.9	36.2	**35.2**	**35.5**	27.4	22.7	19.4	19.0	14.0

1.4	198.8	**146.1**	**108.7**	**76.1**	40.6	17.7	12.7	29.4	30.8	**30.2**	22.1	17.3	13.8	14.8	9.8

1.5	**266.1**	**158.4**	**109.2**	**73.4**	37.3	19.5	12.5	25.9	25.8	24.4	16.4	11.5	8.0	11.7	9.4

^a^International Classification of Childhood Cancer.

^b^Strength, $S=\left(\frac{\left[O-E\right]}{E}\right)\times 100$, counts of pairs that are close in time and space. Cases are close in space if distances between their locations differ by less than 0.5,...,7.5 km. Cases are close in time if dates of diagnosis differ by less than 0.1,...,1.5 year. Nominally statistically significant (*P *< 0.05) values of *S *have been emboldened.

Kulldorff's scan statistic was used to identify individual clusters and found a statistically significant space-time cluster for all CNS tumours based on place and date of birth (occurring during 1982; *O *= 5, *E *= 0.23, *O*/*E *= 22.19, *P *= 0.014), a significant space-time cluster for all CNS tumours based on place and date of diagnosis (occurring during 1975; *O *= 4, *E *= 0.11, *O*/*E *= 36.47, *P *= 0.036) and a marginally significant space-time cluster for PNETs based on place and date of diagnosis (occurring during 1994; *O *= 3, *E *= 0.097, *O*/*E *= 30.94, *P *= 0.096).

Analysis of cases of PNET by gender (based on place and date of diagnosis) showed that there was evidence of overall clustering both for pairs of cases that included at least one male and also for pairs of cases that included at least one female (*P *= 0.02 using the NN threshold approach). A comparison using a Bernoulli-based model (a "case-control" approach) found no significant differences for individual clustered cases between males ("cases") and females ("controls").

Analysis of cases of PNET by level of population density (based on place and date of diagnosis) showed that there was more striking evidence of overall clustering for pairs of cases that included at least one from a "more densely populated area" (*P *= 0.02 using the geographical distance approach and *P *= 0.005 using the NN threshold approach) than for pairs of cases that included at least one from a "less densely populated area" (*P *= 0.18 using the geographical distance threshold and *P *= 0.04 using the NN threshold). However, a comparison using a Bernoulli-based model (a "case-control" approach) found no significant differences between levels of population density for individual clusters cases (where "cases" are from a more densely populated area and "controls" are from a less densely populated area).

## Discussion

This study has found evidence of overall space-time clustering amongst cases of the PNET sub-type (partly supporting *prior hypothesis (i) *that a transient environmental agent may be involved in aetiology). For PNETs, there was no evidence of any difference between males and females, indicating that geographical or temporal heterogeneity of incidence of this childhood CNS tumour is not modulated by differences in susceptibility between males and females (thus *prior hypothesis (ii) *was not supported). However, for PNETs there was some evidence for more striking overall space-time clustering occurring among pairs of cases that included at least one from a "more densely populated area". This suggests that the geographical or temporal heterogeneity of incidence of this childhood CNS tumour was modulated by differences in patterns of exposure related to level of population density and supports *prior hypothesis (iii)*.

This study has the following merits: (i) it is much more up to date than the previous analyses from the whole of GB; (ii) two distance metrics were used: fixed geographical distance thresholds and variable nearest neighbour (NN) thresholds, allowing an assessment to be made to determine whether clustering is more likely to have arisen from a geostationary or an infective process (this was not done in the previous studies from GB [12,13]); (iii) the full set of diagnostic groups were analyzed; and (iv) full diagnostic case review was performed consistently by a single experienced neuropathologist. The analyses were performed using rigorous statistical methods on high-quality population-based incidence data.

If clustering is identified only by the geographical distance method in a heterogeneous population, this could suggest that it has occurred purely as an artefact of variations in population density. However, in this study, evidence of clustering was found using both geographical and NN threshold critical values for distance. Hence, the space-time clustering cannot be attributed to variability of population density. A recent study has demonstrated that Kulldorff's scan statistic correctly assesses the statistical significance of the most likely cluster, but assessment of secondary clusters is more conservative [37]. Since the clusters that were identified were the most likely for each separate analysis, the statistical significance has been correctly assessed.

The *K*-function analyses used two distinctive types of spatial threshold. Space-time clustering based on fixed distance critical values suggests a role for transient but geostationary aetiological factors. In contrast, space-time clustering based on heterogeneous NN thresholds suggests a role for a transient agent that is spread by contact between individuals. Space-time clustering for PNETs was present using both types of threshold. However, there was little difference in the *P*-values between the fixed geographical and NN metrics and so it is not clear whether the clustering was driven by a geostationary or an infective process. Further research will focus on attempts to differentiate between these two processes.

The finding of space-time clustering from the present study is consistent with a transient aetiological agent. Examples of such agents that have been identified from case-control studies include farm exposures, pesticides and insecticides [3,4,7]. Furthermore, findings of seasonality and space-time clustering are consistent with transient exposures such as infections and air pollution [9-13]. It must be stressed that space-time clustering would only occur when the latent period from exposure to diagnosis is short (or at least relatively constant) and that this would only happen for a minority of cases. The *K*-function and Knox analyses are systematic methods for determining the presence of overall space-time clustering, but do not elicit understanding of individual clusters. In addition, the scan statistic analyses identified some evidence of individual space-time clusters. For PNETs, there was evidence of small case aggregations. There was some evidence that space-time clustering of PNETs was more marked in more densely populated areas, which would be consistent both with environmental exposures such as pollution and with more opportunity for person-to-person transmission of an aetiological agent (such as an infection).

A previous study of national GB incidence data on childhood (ages 0-14 years) CNS tumours diagnosed during the period 1969-1993 found space-time clustering amongst all CNS tumours based on place of birth and date of birth, but not for astrocytoma nor for PNETs [13]. Further analysis of the same data set found marginally significant (*P *= 0.06) space-time clustering of astrocytoma based on date of diagnosis and both place of birth and place of diagnosis [12,13]. However, these studies were based on the NN threshold metric only. Another study, of cases diagnosed during the period 1954-1998 from North West England, found evidence of space-time clustering of cases of childhood astrocytoma around both birth and diagnosis [14]. A comparison of the findings from the childhood studies is given in Table 7. A study of adult gliomas from the Netherlands found that there was overall evidence of space-time clustering which could not be attributed to a particular sub-type [15,16]. Our results contrast with previous studies of space-time clustering of CNS tumours: we found evidence of space-time clusters in the overall group and evidence specifically for space-time clustering amongst PNETs. Since space-time clustering of PNETs was still present when two shorter time periods were examined (1974-1990 and 1991-2006), the overall space-time clustering is unlikely to have arisen because of population shifts.

**Table 7:** Summary of findings of positive (+ve)^a ^and negative (-ve)^b ^findings of space-time clustering from recent childhood studies

Study/Disease Group (ICCC^c ^Code(s))	McNally and colleagues^7^	McNally and colleagues^5,6^	This study
Ependymoma (III(a))	-ve	^d^	-ve

Astrocytoma (III(b))	+ve	-ve	-ve

Ependymoma and Astrocytoma (III(a), III(b))	+ve	^d^	-ve

PNET (III(c))	-ve	-ve	+ve

Other gliomas (III(d))	-ve	^d^	-ve

All CNS tumours (III(a-f))	+ve	+ve	-ve

^a^*P *< 0.05.

^b^*P *≥ 0.05.

^c^International Classification of Childhood Cancer.

^d^Not analysed

A number of other epidemiological studies have suggested that infections may be involved in the aetiology of childhood CNS tumours [38-41]. The findings of space-time clustering from this study are consistent with the involvement of infections in aetiology. However, the specific sub-type involved differed from previous studies. Furthermore, the findings are also consistent with other environmental exposures such as pesticides, insecticides and pollution [3,4,7]. Together these findings suggest that the involvement of a transient environmental agent is not specific to a particular sub-type. We would hypothesise that one or more transient environmental agents may act as a trigger precipitating the final event leading to the onset of a tumour.

## Conclusions

The present study updates and extends previous analyses. Rigorous data collection and pathological review procedures have ensured excellent completeness of ascertainment and accurate classification of CNS sub-types. There is some evidence for an environmental component to the aetiology of PNETs. However, a possible role for chance cannot be excluded. Future studies should examine differences between "clustered" and "non-clustered" cases in the types and nature of putative transient environmental agents.

## Competing interests

The authors declare that they have no competing interests.

## Authors' contributions

RJQM, PWJ and RGF contributed to the design of the study, the writing of the manuscript and the analysis and interpretation of data, SVP, PAM and MVL contributed to the writing of the manuscript and the interpretation of data.

## Pre-publication history

The pre-publication history for this paper can be accessed here:

http://www.biomedcentral.com/1471-2407/12/13/prepub
